# Biotherapeutic Effect of Gingival Stem Cells Conditioned Medium in Bone Tissue Restoration

**DOI:** 10.3390/ijms19020329

**Published:** 2018-01-23

**Authors:** Francesca Diomede, Agnese Gugliandolo, Domenico Scionti, Ilaria Merciaro, Marcos FXB Cavalcanti, Emanuela Mazzon, Oriana Trubiani

**Affiliations:** 1 Department of Medical, Oral and Biotechnological Sciences, University “G. d’Annunzio” Chieti-Pescara, 66100 Chieti, Italy; francesca.diomede@unich.it (F.D.); ilaria.merciaro@unich.it (I.M.); trubiani@unich.it (O.T.); 2 IRCCS Centro Neurolesi “Bonino Pulejo”, 98124 Messina, Italy; agnesegugli@hotmail.it (A.G.); domenico.scionti@gmail.com (D.S.); 3 Faculté de Médecine, UMR 7365 CNRS-Université de Lorraine, 9, Avenue de la Forêt de Haye, 54500 Vandoeuvre-lés-Nancy, France; mxistocavalcanti@gmail.com; 4 Laser in Dentistry Program, Cruzeiro do Sul University (UNICSUL), Sao Paulo 08060-070, Brazil

**Keywords:** conditioned medium, gingival fibroblast, biomaterial, tissue regeneration

## Abstract

Bone tissue engineering is one of the main branches of regenerative medicine. In this field, the use of a scaffold, which supported bone development, in combination with mesenchymal stem cells (MSCs), has promised better outcomes for bone regeneration. In particular, human gingival mesenchymal stem cells (hGMSCs) may present advantages compared to other MSCs, including the easier isolation. However, MSCs’ secretome has attracted much attention for its potential use in tissue regeneration, such as conditioned medium (CM) that contains different soluble factors proved to be useful for the regenerative purposes. In this study, we evaluated the osteogenic capacity of a poly-(lactide) (3D-PLA) scaffold enriched with hGMSCs and hGMSCs derived CM and its ability to regenerate bone defects in rat calvarias. 3D-PLA alone, 3D-PLA + CM or 3D-PLA + hGMSCs with/without CM were implanted in Wistar male rats subjected to calvarial defects. We observed that 3D-PLA scaffold enriched with hGMSCs and CM showed a better osteogenic capacity, being able to repair the calvarial defect as revealed in vivo by morphological evaluation. Moreover, transcriptomic analysis in vitro revealed the upregulation of genes involved in ossification and regulation of ossification in the 3D-PLA + CM + hGMSCs group. All of these results indicate the great osteogenic ability of 3D-PLA + CM + hGMSCs supporting its use in bone regenerative medicine, in particular in the repair of cranial bone defects. Especially, hGMSCs derived CM played a key role in the induction of the osteogenic process and in bone regeneration.

## 1. Introduction

Bone is a mineralized connective tissue with important functions, including locomotion, support and protection of soft tissues, calcium and phosphate storage. It is highly dynamic being always resorbed by osteoclasts and neoformed by osteoblasts [1]. In some conditions, bone regeneration is required in large quantity, such as in the case of large bone defects caused by trauma, degeneration or skeletal abnormalities, or when regenerative process is compromised. In this case, bone regeneration represents an important problem and there is a need for treatments. In this context, bone tissue engineering has promised good results.

The use of a scaffold with cells and/or soluble factors has emerged as a promising approach for bone regeneration in the field of regenerative medicine. A biomaterial refers to a matrix that provides a specific environment and support for bone growth and development. An ideal scaffold must be biocompatible and non-toxic, have good osteoinductivity and osteoconductivity and should improve cell viability, cell adhesion, proliferation and osteogenic differentiation [2,3]. Different biomaterials were used for bone repair, such as poly-(lactide) (PLA). PLA is a hydrophobic aliphatic polyester with various biomedical and clinical applications [4]. PLA is one of the most used biomaterials in different fields of regenerative medicine, thanks to its properties including its biocompatibility and non-toxicity of both PLA and its degradation products [5]. In particular, PLA was used for the construction of scaffolds for bone and osteochondral regeneration [6,7].

Scaffolds can be combined with cells, such as mesenchymal stem cells (MSCs) that can promote bone regeneration through the differentiation towards the osteogenic lineage or releasing specific soluble factors, or scaffolds can be primed with soluble molecules, including growth factors that can be delivered in the environment performing a therapeutic action [3]. 

MSCs have attracted much attention in regenerative medicine. They can be isolated from different tissues, such as bone marrow, umbilical cord, adipose tissue and dental tissues, and they have a multilineage differentiation potential [8]. In particular, the ability to differentiate into osteoblasts is one of the minimal criteria to identify MSCs [9]. Even if bone marrow MSCs are the most studied, other sources may present advantages, such as dental derived MSCs. Indeed, they are easier to isolate and have promised good results in regenerative medicine, including bone regeneration [10,11,12]. Among oral derived MSCs, gingival MSCs (GMSCs) showed a great capacity in bone reconstruction and repair for clinical use [13,14]. GMSCs present different advantages, including that their collection did not require invasive procedures and allow MSCs collection from the patient for autologous transplantation. Moreover, they had different advantages compared to bone marrow-derived MSCs. Indeed, GMSCs are easy to isolate, homogenous and proliferate faster than bone marrow MSCs, present a stable morphology, maintain a normal karyotype and MSC characteristics also after long-term cultures [15]. Interestingly, periodontally affected dental tissue may be used for the collection of MSCs; indeed, cells maintain both stem cell properties and the osteogenic ability [16]. However, it was speculated that the main effects of MSCs may be mediated by a paracrine mechanism. It is known that MSCs release different factors, including growth factors, soluble proteins, free nucleic acids, also called the secretome of MSCs [17]. In particular, the conditioned medium (CM) contains different growth factors secreted by MSCs, which may be useful for the regenerative purposes, and showed good results in different diseases [18]. It was reported that CM derived from bone marrow MSCs was useful for bone regeneration, thanks to its content in cytokines [19,20,21,22]. Interestingly, the effects of CM on bone regeneration seem to be stronger than those of MSCs [19]. Bone regenerative capacity was reported also for CM derived from adipose MSCs [23].

The aim of this study was the investigation of the osteogenic potential of a 3D-PLA scaffold enriched with human GMSCs (hGMSCs) and CM derived from hGMSCs in vitro and in vivo. In particular, given the reported beneficial effects of CM on bone regeneration, we investigated whether the enrichment with hGMSCs derived CM improved the osteogenic performance of the biological construct. To our knowledge, the osteogenic ability of hGMSCs derived CM has never been investigated. With this aim, we evaluated the transcriptional profile by next generation sequencing (NGS) and the osteogenesis performance in vitro of 3D-PLA scaffold alone and 3D-PLA scaffold enriched with hGMSCs and/or CM. Moreover, their capacity to regenerate bone defects induced in rat calvarias was investigated.

## 2. Results

### 2.1. Human GMSCs Characterization

**Figure 1:**
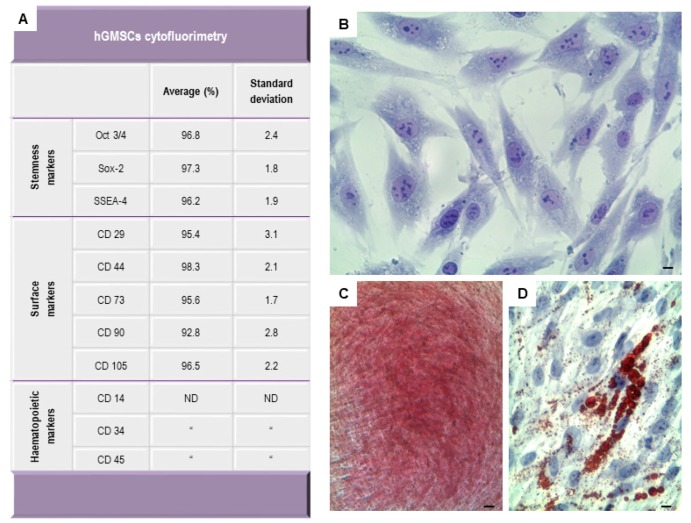
Human gingival mesenchymal stem cells (hGMSCs) characterization. (**A**) flow cytometry showed the positivity expression of Oct3/4, Sox-2, SSEA-4, CD29, CD44, CD73, CD90 and CD105. While haematopoietic markers are not detectable; (**B**) light inverted microscopy image of plastic-adherent hGMSCs stained with toluidine blue solution; (**C**) hGMSCs under osteogenic differentiation conditions stained with alizarin red S solution; (**D**) hGMSCs stained with Adipo Oil red to demonstrate adipogenic differentiation. Scale bars represent 10 μm.

The hGMSCs, isolated from gingival tissue were positive for several antigens, as Oct3/4, Sox-2, SSEA-4, CD29, CD44, CD73, CD90 and CD105, while lacking in the expression of hematopoietic surface molecules as CD34, CD14 and CD45 (Figure 1A). hGMSCs were plastic-adherent cells (Figure 1B) and capable of extensive proliferation when maintained in their standard culture conditions; moreover, they are able to differentiate into mesengenic lineages. In hGMSCs cultures, the Alizarin Red staining highlighted the presence of calcium deposition in osteogenic differentiation medium-treated cells after three weeks of induction (Figure 1C). Oil Red O positive lipid droplets were evident in the cytoplasm of hGMSCs induced to adipogenic commitment after 28 days of induction (Figure 1D). 

### 2.2. 3D-PLA Scaffold Characterization 

**Figure 2:**
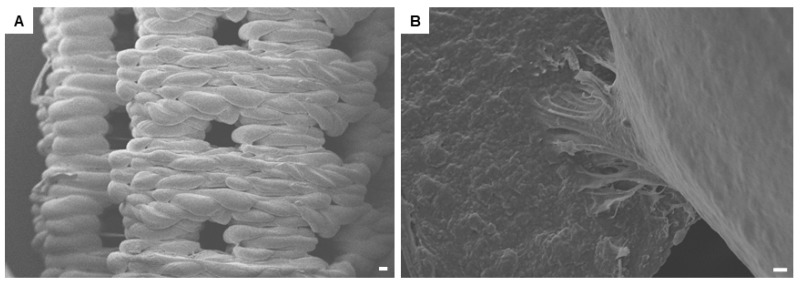
Scanning electron microscope (SEM) detection. (**A**) three-dimensional scaffold design observed at low magnification (50×). The scale bar represents 100 μm; (**B**) at high magnification (1000×), the cellular bridge is clearly visible. The scale bar represents 10 μm.

The three-dimensional (3D) structural morphology of printed PLA scaffold is characterized by means of scanning electron microscopy (SEM) (Figure 2A). Surface morphology is represented by a modular structure. At high magnification, hGMSCs adhesion onto the substrate is observable. Cells developed a bridge-like structure on the PLA filaments (Figure 2B). 

### 2.3. Osteogenic In Vitro Performance

**Figure 3:**
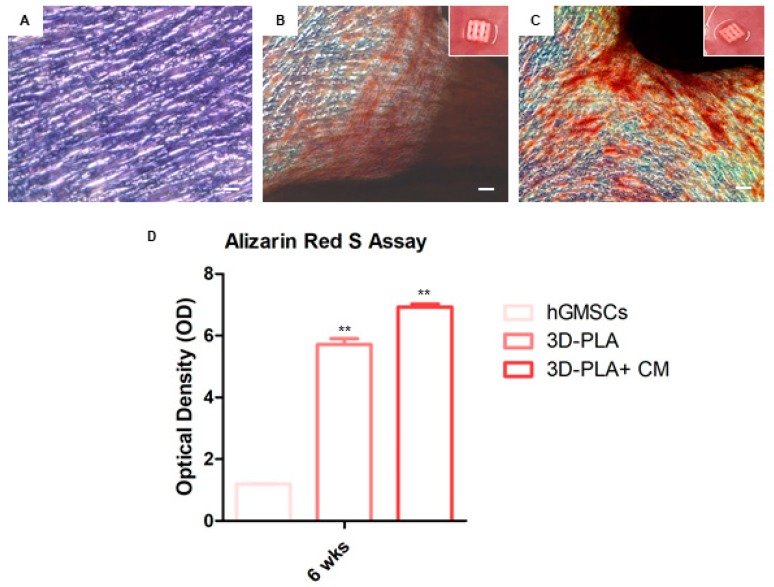
In vitro osteogenic performance. hGMSCs maintained under standard conditions for six weeks and subsequently stained with Alizarin Red S solution. (**A**) hGMSCs used as control culture; (**B**) hGMSCs cultured with poly-(lactide) (3D-PLA) scaffold; (**C**) hGMSCs cultured with 3D-PLA + conditioned medium (CM); (**D**) histograms of alizarin red S staining quantification. Scale bars represent 10 μm. Graph optical density: ** *p* < 0.01 was considered statistically significant.

hGMSCs were observed by inverted light microscopy to verify the alizarin red S staining (Figure 3). Microphotographs showed the red positive staining in 3D-PLA + CM + hGMSCs when compared with 3D-PLA + hGMSCs. Insets in Figure 3B,C are a representative macrophotographs of 3D-PLA placed in multiwell cell culture plates. Obtained results were evaluated using spectrometric analysis after six weeks of culture (Figure 3D). 

### 2.4. Global Transcriptome Analysis

**Figure 4:**
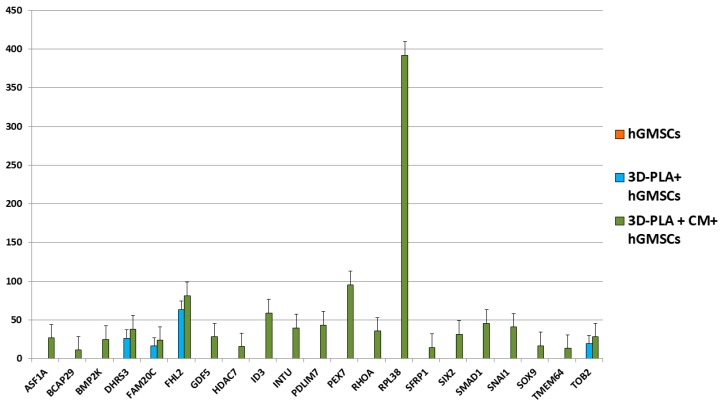
Expression levels of genes differentially expressed in hGMSCs, 3D-PLA + hGMSCs and 3D-PLA + conditioned medium (CM) + hGMSCs (false discovery rate (FDR) < 0.05; *n* = 3).

The transcriptome of hGMSCs, 3D-PLA + hGMSCs and 3D-PLA + CM + hGMSCs was investigated through NGS analysis. We observed that, among the genes that were differentially expressed between these groups, 21 genes were totally inhibited or expressed at very low levels in hGMSCs, while they were expressed in 3D-PLA + hGMSCs and/or 3D-PLA + CM + hGMSCs. Interestingly, the gene ontology (GO) analysis of these genes revealed that eight genes belonged to the category ossification, eight genes to the category regulation of ossification, while five genes were in common for both categories. The analysis showed that the genes BCAP29 and BMP2K were slightly downregulated in 3D-PLA + hGMSCs compared to hGMSCs. Instead, these two genes were upregulated in 3D-PLA + CM + hGMSCs compared to the other groups. The genes DHRS3, FAM20C, FHL2 and TOB2 were upregulated in 3D-PLA + hGMSCs compared to hGMSCs (Figure 4). In addition, the expression levels of these genes were upregulated in 3D-PLA + CM + hGMSCs compared to 3D-PLA + hGMSCs (Figure 4). Instead, the genes ASF1A, GDF5, HDAC7, ID3, INTU, PDLIM7, PEX7, RHOA, RPL38, SFRP1, SIX2, SMAD1, SNAI1, SOX9 and TMEM64 were expressed only in 3D-PLA + CM + hGMSCs (Figure 4). 

Gene expression levels, fold changes, gene ontology (GO) processes and false discovery rate (FDR) were reported in Table 1.

### 2.5. 3D-PLA In Vivo Evaluation

Bone regeneration in the implanted areas was evaluated at six weeks after surgery. Macroscopic evaluation showed a better regeneration capacity of 3D-PLA + CM + hGMSCs. 

**Figure 5:**
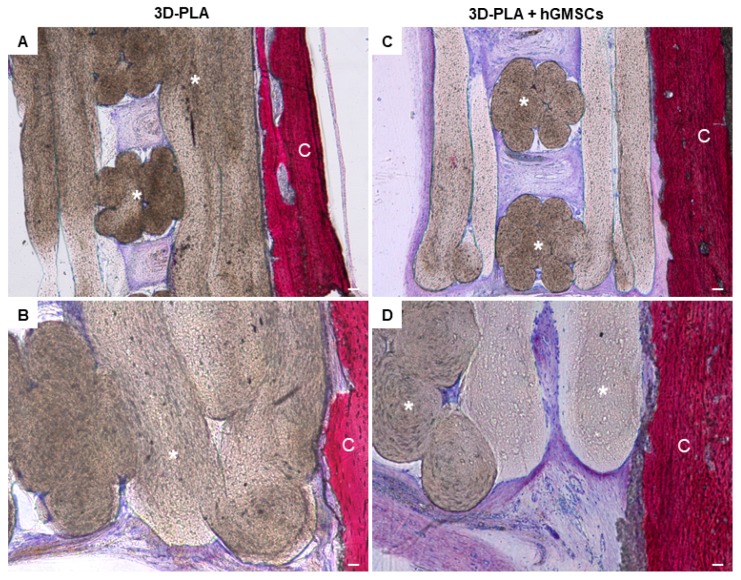
Histological examination of 3D-PLA group and 3D-PLA + hGMSCs group. Representative methylene blue and acid fuchsin images. (**A**) 3D-PLA group at low magnification (4×); (**B**) 3D-PLA group at high magnification (20×); (**C**) 3D-PLA + hGMSCs group at low magnification (4×); (**D**) 3D-PLA + hGMSCs group at high magnification (20×). Scale bars represent 10 μm. *: 3D-PLA scaffold; C: rat calvaria.

In the 3D-PLA scaffold group, the growing woven extracellular matrix (ECM) not fully covered the interstitial area of the biomaterial porous at six weeks after surgery (Figure 5A). At high magnification, empty areas are visible inside the biomaterial structure (Figure 5B). The 3D-PLA + hGMSCs group showed more ECM deposition inside the biomaterial scaffold when compared to the 3D-PLA group (Figure 5C). At high magnification ECM, without signs of mineralization, is present on the filaments side and at the interface of the native bone and scaffold (Figure 5D).

**Figure 6:**
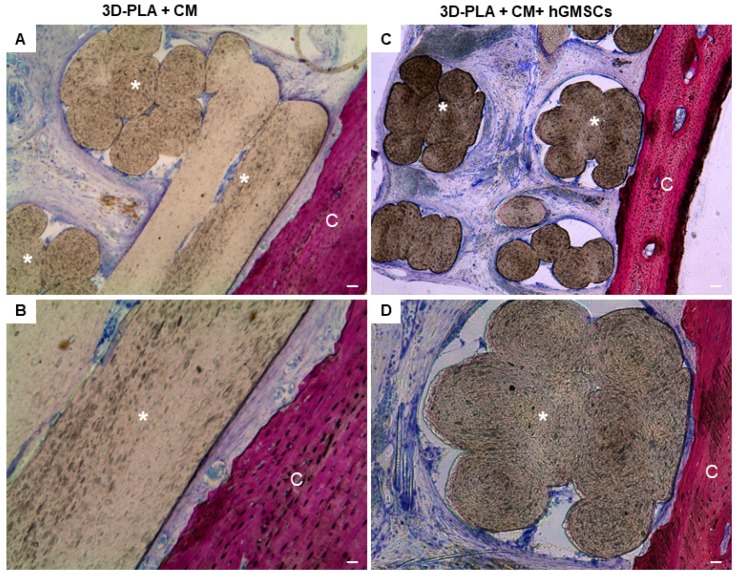
Histological examination of 3D-PLA + CM group and 3D-PLA + CM + hGMSCs group. Representative methylene blue and acid fuchsin images. (**A**) 3D-PLA + CM group at low magnification (4×); (**B**) 3D-PLA + CM group at high magnification (20×); (**C**) 3D-PLA + CM + hGMSCs group at low magnification (4×); (**D**) 3D-PLA + CM + hGMSCs group at high magnification (20×). *: 3D-PLA scaffold; C: rat calvaria. Scale bars represent 10 μm.

In the 3D-PLA + CM group, a strong ECM deposition inside the biomaterial structure and on the native bone tissue was visible (Figure 6A). At high magnification, different new blood vessel structures are visible in the ECM and on the interface of scaffold and host tissue, in order to indicate the beginning of the regeneration process (Figure 6B). In contrast, less blood vessels are visible in 3D-PLA + CM + hGMSCs (Figure 6C,D).

A summary of these results is reported in Table 2.

## 3. Discussion

Materials for tissue engineering should possess specific features in order to be considered as ideal material for grafting in bone tissue. Mainly, they should be biocompatible, bioactive, and act as a 3D template for cellular activity in order to be well integrated with the surrounding biological environment of the implant site and to trigger specific cellular responses [24]. 3D printing is becoming an increasingly common technique to fabricate scaffolds and devices for tissue engineering applications that were previously difficult to build [25]. A 3D-printing technique for medical devices has become a popular tool to develop scaffolds for clinical use, and they can be customized, fabricated feasibly and economically advantageous [26]. PLA is an absorbable polymer that has often been used in skeletal tissue engineering that ensure mechanical stability while degrading. The degradation products of this polymer are non-toxic and removed by natural metabolic pathways [27].

In our study, thick PLA scaffolds with square pores were produced by a custom-made 3D printer based on this principle. The printed scaffolds were then functionalized with hGMSCs and/or CM and then evaluated for their capacity to induce in vitro an osteogenic process and in vivo to start the osteogenic repair of bone defects when grafted in rat calvaria. 

Actually, MSCs have been applied for autologous therapy in combination with platelet-rich plasma and/or scaffolds in distraction osteogenesis. In particular, therapeutic ability of MSCs has been attributed at least to three major mechanisms: (1) homing process, whereby the systemic stem cell delivery results in cell migration to specific areas of acute injury via chemical gradients; (2) differentiating process, in order to locally regenerate or replace the damaged tissues; and (3) secreting bioactive molecules, in order to affect local and systemic processes via paracrine mechanisms [28,29].

Human GMSCs have recently been proposed as a novel option in regenerative therapy. They express MSC surface markers, such as Oct3/4, Sox-2, SSEA-4, CD29, CD44, CD73, CD90 and CD105, and lacking the expression for CD34, CD14 and CD45 [30]. Moreover, they are able to differentiate into adipogenic and osteogenic lineage as demonstrated by biochemical analysis and possess a canonical fibroblast like morphology. hGMSCs are thought to be good candidate cell sources, easy to access and manipulate, in order to use in tissue engineering [31]. A recent study reported that the oral MSCs cultured onto 3D-scaffolds grafted in the mouse calvaria lead an osteointegration and vascularisation process.

Previously, we have demonstrated that the PLA scaffolds are non-cytotoxic and biocompatible. In our experiments, SEM analysis revealed that hGMSCs are able to adhere and grown onto the scaffold, covering the biomaterial surface without any morphological changes in cytoskeleton rearrangement. Indeed, PLA showed a good biocompatibility with another MSC type derived from oral tissues and the stem cells from human exfoliated deciduous teeth (SHED), inducing also the mineralization of SHED [32]. In addition, a star-shaped PLA was reported to induce the differentiation of human adipose MSCs toward the osteogenic lineage in vitro and enhanced the mineralized tissue formation in vivo [33]. These features, in accordance with our results, indicated that PLA is a good biomaterial for bone tissue engineering.

To improve the performance of the PLA scaffold in our study, we evaluated the effects of the combination of the scaffold with the CM and hGMSCs. MSCs possess immunomodulatory and anti-inflammatory properties and using paracrine signaling, they can exert their action in the implanted site and favorably affect in vitro the extracellular matrix remodeling [34,35]. The secretome of MSCs contains many growth factors, such as insulin growth factor that can affect the migration, angiogenesis, and osteogenic differentiation and possess a great potential effect for bone and periodontal tissue regeneration [35]. CM derived from human adult MSCs has been already applied to accelerate bone formation in preclinical animal models [36]. Osugi and co-workers [19] showed that CM derived from human bone marrow–MSCs, containing different cytokines including insulin-like growth factor-1 and vascular endothelial growth factor, enhanced the migration, proliferation, and expression of osteogenic marker genes in rat MSCs in vitro. In addition, CM increased bone regeneration in vivo in rats with calvaria defects, and, in particular, after eight weeks, the new regenerated bone nearly covered the defect. In particular, it seems that CM improved bone regeneration through the mobilization of endogenous MSCs [19].

The results from our study suggest that the 3D-PLA + CM + hGMSCs might improve the osteogenic process in vitro as demonstrated by alizarin red staining; indeed, calcium depositions are more evident in the presence of CM when compared with the other experimental groups. Thus, the utilization of the CM not only might halt bone loss in diseases such as periodontitis, but also could improve the quality of the new bone with increased mineralization and connectivity. 

The transcriptome profile showed that 21 genes were inhibited or expressed at low levels in hGMSCs, but expressed in 3D-PLA + hGMSCs and/or 3D-PLA + CM + hGMSCs. hGMSCs expressed only the genes BCAP29 and BMP2K at very low levels. 3D-PLA + hGMSCs showed a slight downregulation of these two genes, while the other four genes were expressed and upregulated compared to hGMSCs. All the 21 genes were expressed in 3D-PLA + CM + hGMSCs. In particular, the expression of all these genes was upregulated in 3D-PLA + CM + hGMSCs compared to the other groups. Interestingly, GO analysis revealed that these genes belonged to the categories involved in ossification and regulation of ossification. Then, NGS analysis showed that the hGMSCs grown on the 3D-PLA scaffold enriched with the CM expressed osteogenic genes compared to hGMSCs and 3D-PLA + hGMSCs, suggesting that the CM plays a main role in the induction of an osteogenic transcriptional program. 

In earlier in vivo studies, 3D-PLA has demonstrated a well integration with the host tissue. Our in vivo results confirmed the main role played by CM in bone regeneration. Indeed, in the 3D-PLA group, ECM not fully covered the interstitial area of the biomaterial, while the 3D-PLA + hGMSCs group showed more ECM deposition inside the biomaterial scaffold but without signs of mineralization. Instead, the histological analysis of bone regeneration in the calvarial defects revealed improvement of new bone formation in the group implanted with 3D-PLA + CM as reflected by the increase in ECM formation and bone contact when compared to the other groups by the six weeks time-point. Moreover, the macroscopic evaluation showed a better bone repair in 3D-PLA + CM + hGMSCs group.

We can suggest that the implant of 3D-PLA enriched with both CM and hGMSCs may present some advantages. First, cytokines and growth factors contained in the CM could activate an osteogenic differentiation program in hGMSCs, increasing osteogenic gene expression and mineralization as demonstrated by our in vitro results. Given that it has already been demonstrated that GMSCs and CM participated in bone repair, also recruiting bone progenitor cells and endogenous MSCs [13,19], we can suggest that in vivo CM may act inducing the mobilization and then the osteogenic differentiation of both hGMSCs and endogenous MSCs. Second, a problem of stem cell therapy is the poor viability of cells after the implant. However, the presence of the scaffold and the CM could increase the viability rate of hGMSCs. Third, hGMSCs in situ may continue to release factors useful for the regenerative process. 

The porous 3D-PLA scaffold can promote the exchange of body fluid and vascularization, but the CM could have a key role in the osteoblasts recruiting in the surrounding tissues stimulating the synthesis and accumulation of ECM, leading to an enhanced osteoid deposition [37]. Our data are therefore in agreement with the promising results obtained on the use of CM that accelerates the formation of new bone callus stimulating the recruitment of endogenous bone marrow derived-MSCs [36]. To our knowledge, this is the first study to evaluate the effects of CM derived from hGMSCs in bone tissue regeneration. Given that hGMSCs present advantages compared to other MSCs, such as easy isolation, the fact that their CM shows efficacy in tissue regeneration, encourage CM deep characterization in order to allow its use in the clinical practice in the future.

## 4. Materials and Methods 

### 4.1. Cell Culture and Characterization

Written approval for gingival biopsy collection was obtained from the Medical Ethics Committee at the Medical School, “G. d’Annunzio” University, Chieti, Italy and each participant gave informed consent. Gingival tissue biopsies were obtained from healthy adult volunteers with no gingival inflammation as previously described by Diomede et al. [38]. To define the surface molecules flow citometry was performed as previously reported [39]. Plastic adherent cells were stained with toluidine blue solution and to evaluate cell morphology were observed at light microscopy (Leica, DMIL, Milan, Italy) [40]. To evaluate the capacity to differentiate into mesengenic lineages, cells were cultured under specific culture conditions, osteogenic and adipogenic respectively as reported by Ballerini et al. [41].

### 4.2. Scaffold Development and Three-Dimensional Scaffold Constructs 

The scaffold was obtained from a commercial poly-(lactide) (PLA) (Kaytech srl, Ancona, Italy). Samples were developed with a commercial CAD software (Rhinoceros 5, McNeel Europe, Barcelona, Spain) as previously described; the projects were then applied to a printing slicing software (Cura 15.04, Ultimaker B.V., Geldermalsen, The Netherlands). The sliced project was finally transferred to a commercial fuse filament fabrication 3D printer (DeltaWASP 2040; CSP srl, Massa Lombarda, Italy) [42].

### 4.3. Scanning Electron Microscopy (SEM) Characterization 

A Scanning Electron Microscope (EVO 50 XVP; Zeiss, Jena, Germany) was used to image the surfaces of the scaffold; the specimens were sputtered with 4–8 nm of gold and then mounted on carbon tape dots [43]. 

### 4.4. Conditioned Medium (CM) Collection

The CM, after 72 h of incubation, was collected from 15 × 10^3^/cm^2^ hGMSCs at the 2nd passage. The CM was centrifuged at 1200 rpm for 5 min to eliminate suspension cells and debris. The supernatants were recentrifuged at 3000 rpm for 3 min, followed by collection of the secondary supernatants. Subsequently, 1 mL of secondary supernatants was resuspended in 3 mL of ice aceton and maintained over night at 4 °C, and after centrifuged at 16.000 rpm for 12 min at 4 °C (Centrifuge 5804 R, Eppendorf, Milan, Italy). The suspension was lysated in radioimmunoprecipitation assay (RIPA) buffer and quantified by means Breadford assay [34,44]. Total proteins obtained were 125 µg/µL.

### 4.5. In Vitro Osteogenesis Performance

hGMSCs were cultured at 8 × 10^3^ cells/cm^2^ in standard culture medium (MSCGM-CD) (Lonza, Basilea, Switzerland). 3D-PLA was pre-treated for 24 h under agitation (MacsMix, Milthenyi, Bologna, Italy) with 5 mL of CM. Evaluation of calcium deposition and ECM mineralization was obtained by Alizarin Red S (ARS) staining assay performed after 6 weeks. Cells were washed with PBS, fixed in 10% (*v*/*v*) formaldehyde (Sigma-Aldrich, Milan, Italy) for 30 min and washed twice with abundant dH_2_O prior to addition 0.5% Alizarin red S in H_2_O, pH 4.0, for 1 h at room temperature. After cell incubation under gentle shaking, cells were washed with dH_2_O four times for 5 min. For staining quantification, 800 μL 10% (*v*/*v*) acetic acid was added to each well. Cells incubated for 30 min were scraped from the plate, transferred into a 1.5 mL vial and vortexed for 30 s. The obtained suspension, overlaid with 500 μL mineral oil (Sigma-Aldrich), was heated to 85 °C for 10 min, then transferred to ice for 5 min, carefully avoiding opening of the tubes until fully cooled, and centrifuged at 20,000× *g* for 15 min. In addition, 500 μL of the supernatant were placed into a new 1.5 mL vial and 200 μL of 10% (*v*/*v*) ammonium hydroxide was added (pH 4.1–pH 4.5). Furthermore, 150 μL of the supernatant obtained from cultures were read in triplicate at 405 nm by a spectrophotometer (Synergy HT, BioTek, Bad Friedrichshall, Germany). 

### 4.6. RNA Extraction

Total RNA was isolated from hGMSCs, 3D-PLA + hGMSCs and 3D-PLA + CM + hGMSCs cultured for 1 week using the Total RNA Purification Kit (Norgen Biotek Corp., Ontario, CA, USA) according to the manufacturer’s protocol. Total RNA was quantified by means BioSpectrometer (Eppendorf, Milan, Italy) using μCuvette G1.0 (Eppendorf, Milan, Italy). 

### 4.7. RNA Sequencing and Library Preparation

RNA sequencing libraries were obtained using the TruSeq RNA Access library kit (Illumina, Inc., San Diego, CA, USA) following the manufacturer’s instructions. In summary, RNA (50 ng) of each sample was fragmented at 94 °C for 8 min. The synthesis of first strand cDNA was carried out using random hexamers and the SuperScript II Reverse Transcriptase (Invitrogen, Milan, Italy) at 25 °C for 10 min, 42 °C for 15 min, and 70 °C for 15 min.

In the second strand cDNA synthesis, the RNA templates were removed and a second replacement strand was synthesized by dUTP internalization, generating a double strands cDNA. In order to purify the blunt-ended-cDNA, AMPure XP beads (Beckman Coulter, Brea, CA, USA) were used. Afterwards, 3′ ends of the cDNA were adenylated to allow the adaptor ligation in the following step. After adaptor ligation, the libraries were purified with AMPure XP beads and a first PCR amplification step was carried out in order to enrich those DNA fragments with adaptors on both ends but also to increase the amount of DNA in the library (15 cycles of 98 °C for 10 s, 60 °C for 30 s, and 72 °C for 30 s). After library validation, 200 ng of each DNA library was combined and the first hybridization step was performed using exome capture probes according to a standardized protocol at the following conditions: 18 cycles of 1 min of incubation, starting at 94 °C, and then decreasing by 2 °C for every cycle. Magnetic beads coated with streptavidin were used to capture probes hybridized to the target regions. Then, the enriched libraries were eluted from the beads and a second cycle of hybridization was performed to obtain a wide specificity of regions of capture. Afterwards, a second capture step using streptavidin-coated beads was performed and two heated washes were done in order to remove nonspecific binding from the beads. After that, the libraries were purified through the AMPure XP bead, and amplified following the protocol (10 cycles; incubation at 98 °C for 10 s, incubation at 60 °C for 30 s and incubation at 72 °C for 30 s), followed by a purification step. Libraries were quantified by qPCR using KAPA Library Quantification Kit—Illumina/ABI Prism (Kapa Biosystems, Inc., Wilmington, MA, USA) and validated with the Agilent High Sensitivity Kit on a Bioanalyzer (Cernusco sul Naviglio, Italy). The size of the DNA fragments has been set in a range of 200–650 bp and peaked around 250 bp. Libraries were normalized to 16 pM and subjected to cluster, and single read sequencing was executed for 300 cycles on a MiSeq instrument (Illumina), according to the protocol guidelines. The produced libraries were loaded for clustering on a MiSeq Flow Cell v2 and sequenced with a MiSeq Instrument (Illumina). The validation of the cluster density was performed by the software of the instrument throughout the run.

### 4.8. NGS Data Processing

Data obtained by NGS analysis were analysed. In particular, the sequence reads were subjected to the demultiplexing process in order to have a separation of the sequence reads in different files for each index tag/sample by using the CASAVA algorithm (CASAVA, version 1.8.2; Illumina, Inc., San Diego, CA, USA). In order to perform the alignment of the sequences, the RNA-Seq Alignment version 1.0.0 (Illumina) and the reference sequence “Homo sapiens University of California, Santa Cruz (UCSC) hg19” were used. For the Read mapping, the TopHat 2 (Bowtie 1) was used. The fragments per kilobase of exon per million fragments mapped (FPKM) values were calculated for each sample using the normalized read counts for each annotated gene: ([1000 × read count] ÷ [number of gene covered bases × number of mapped fragments in million]). Unmapped reads were deleted, preserving only read pairs with both reads aligned to the reference sequence “Homo sapiens UCSC hg19”. The comparison between two different samples was carried out by a scatter plot of the LOG2 of the FPKM.

### 4.9. Statistical Analysis

Statistical analysis was accomplished using ANOVA and Tukey’s post hoc analysis (*p* < 0.05). The statistical analysis on the read counts was carried out with the Cufflinks Assembly and DE package version 2.0.0 to establish the proportion of differentially expressed genes for a *q*-value (FDR) < 0.05. The GO analysis of the genes differentially expressed between experimental group were performed by free tools “Gene Ontolology Consortiun” (available online at http://www.geneontology.org/).

### 4.10. Animals

Male Wistar rats weighing 300–350 g were used for this experiment. Animals were acquired from Harlan, Milan, Italy and housed in individually ventilated cages and maintained under 12 h light/dark cycles, at 21 ± 1 °C and 50–55% humidity with food and water ad libitum.

### 4.11. Ethics Statement for Animal Use 

All animal care and use was accomplished according to the European Organization Guidelines for Animal Welfare. The study has been authorized by the Ministry of Health “General Direction of animal health and veterinary drug” (Authorization 768/2016-PR 28/07/2016-D.lgs 26/2014). The experiments were planned in such a way to minimize the total number of rats needed for the study.

### 4.12. Scaffold Grafting

To implant the scaffold, rats were first anesthetized with a combination of tiletamine and xylazine (10 mL/kg, intraperitoneal; i.p.). Afterwards, the implant site was prepared with iodopovinone (Betadine) after trichotomy. Following a median sagittal incision of about 1.0 cm in the frontoparietal region, a total thickness cut was applied; the calvary was then exposed in the frontal area and in the parietal areas. The circular section bone receiving site, with a diameter of 5 mm and a height of 0.25 mm, was injured by means of a rotary instrument at a controlled speed (trephine milling machine, Alpha Bio-Tec, HTD Consulting S.r.l., Siena, Italy) under constant irrigation of a physiological solution.

For their texture and flexibility, the following 3D-PLA, 3D-PLA + hGMSCs, 3D-PLA + CM, and 3D-PLA + CM + hGMSCs were easily inserted in contact with bone tissue to cover the damaged area. The skin flap was then sutured with Caprosyn 6-0 synthetic monofilament absorbable sutures (Covidien AG, Neuhausen am Rheinfall, Switzerland), using interrupted points. Standard feeding and hydration were maintained constant throughout the post-operative phase. 

### 4.13. Experimental Design

Rats were randomly distributed into the following groups (*N* = 16 total animals):

- 3D-PLA Group (*N* = 4): rats subjected to scraping of the cortical calvaria bone tissue and implant of 3D-PLA;
- 3D-PLA + hGMSCs (*N* = 4): rats subjected to scraping of the cortical calvaria bone tissue and implant of 3D-PLA + hGMSCs;
- 3D-PLA + CM (*N* = 4): rats subjected to scraping of the cortical calvaria bone tissue and implant of 3D-PLA + CM;
- 3D-PLA + CM + hGMSCs (*N* = 4): rats subjected to scraping of the cortical calvaria bone tissue and implant of 3D-PLA + CM + hGMSCs.

After 6 weeks, the animals were euthanized by intravenous administration of Tanax (5 mL/kg body weight) and their calvariae were processed for morphological analysis. 

The specimens were fixed for 72 h in 10% formalin solution, dehydrated in ascending graded alcohols and embedded in LR White resin (Sigma-Aldrich). After polymerization, undecalcified oriented cut sections of 50 μm were prepared and ground down to about 30 μm by using the TT System (TMA2, Grottammare, Italy). 

The sections before to be stained, were analysed with the CLSM (LSM510 META, Zeiss) while, after double staining procedure with methylene blue and fuchsine acid solutions they were observed under a light microscope. 

The investigation was carried out by means of a bright-field light microscope (Leica Microsystem) connected to a high-resolution digital camera DFC425B Leica (Leica Microsystem) [45]. Three-dimensional reconstruction has been obtained by means ZEN2 software (Zeiss). Data and statistical analysis were performed by means the Statistical Package for Social Science (SPSS, v.21.0, IBM Analytics, Armonk, NY, USA). 

## 5. Conclusions

Overall, our data show that the 3D-PLA + CM + hGMSCs induced the formation of new bone and the osseointegration process in the grafted site. Our results confirm previous studies that indicated the utility of the PLA scaffold for bone regeneration. In addition, both in vitro and in vivo results indicated that CM produced by hGMSCs plays a key role in the osteogenic process and in bone regeneration. This is an important result because the collection of hGMSCs is easier compared to other MSCs. Then, CM production from these cells is also easier and less expensive and may cause less problems to the patients. We believe that our findings encourage the use of biofunctionalized scaffold and CM characterization to promote bone regeneration.

## Figures and Tables

**Table 1:** Gene expression levels, fold changes, gene ontology (GO) and false discovery rate (FDR) of differentially expressed genes in human gingival mesenchymal stem cells (hGMSCs), poly-(lactide) (3D-PLA) scaffold + hGMSCs and 3D-PLA + conditioned medium (CM) + hGMSCs.

Gene ID (Entrez)	Name	Description	GO Processes	Gene Expression Value hGMSCs	Gene Expression Value 3D-PLA + hGMSCs	Gene Expression Value 3D-PLA + CM + hGMSCs	Log2 (Fold Change) hGMSCs vs. 3D-PLA + hGMSCs	Log2 (Fold Change) hGMSCs vs. 3D-PLA + CM + hGMSCs	FDR hGMSCs vs. 3D-PLA + hGMSCs	FDR hGMSCs vs. 3D-PLA + CM + hGMSCs
25842	ASF1A	Anti-silencing function 1A histone chaperone	Ossification	0.00	0.00	26.45	0.00	34.62	1.24 × 10^−4^	1.24 × 10^−4^
55973	BCAP29	B-cell receptor associated protein 29	Ossification	0.0047	0.0043	10.94	−0.13	11.17	1.24 × 10^−4^	1.24 × 10^−4^
55589	BMP2K	BMP2 inducible kinase	Regulation of ossification	0.0007	0.0006	24.75	−0.14	15.02	1.24 × 10^−4^	1.24 × 10^−4^
9249	DHRS3	Dehydrogenase/reductase 3	Regulation of ossification	0.00	26.03	38.03	34.60	35.15	1.34 × 10^−3^	3.46 × 10^−4^
56975	FAM20C	Golgi associated secretory pathway kinase	Regulation of ossification	0.00	16.20	23.66	33.91	34.46	1.24 × 10^−4^	3.46 × 10^−4^
2274	FHL2	Four and a half LIM domains 2	Ossification	0.00	63.31	81.21	35.88	36.24	1.46 × 10^−2^	1.24 × 10^−4^
8200	GDF5	Growth differentiation factor 5	Ossification	0.00	0.00	27.97	0.00	34.70	1.24 × 10^−4^	4.00 × 10^−3^
51564	HDAC7	Histone deacetylase 7	Regulation of ossification	0.00	0.00	15.34	0.00	33.84	2.44 × 10^−2^	2.38 × 10^−4^
3399	ID3	Inhibitor of DNA binding 3, HLH protein	Regulation of ossification	0.00	0.00	58.82	0.00	35.78	1.24 × 10^−4^	1.24 × 10^−4^
27152	INTU	Inturned planar cell polarity protein	Regulation of ossification	0.00	0.00	39.70	0.00	35.21	1.24 × 10^−4^	1.24 × 10^−4^
9260	PDLIM7	PDZ and LIM domain 7	Ossification; Regulation of ossification	0.00	0.00	43.41	0.00	35.34	1.24 × 10^−4^	3.86 × 10^−2^
5191	PEX7	Peroxisomal biogenesis factor 7	Ossification	0.00	0.00	95.30	0.00	36.47	2.38 × 10^−4^	1.15 × 10^−3^
387	RHOA	Ras homolog family member A	Ossification	0.00	0.00	35.48	0.00	35.05	2.16 × 10^−3^	1.24 × 10^−4^
6169	RPL38	Ribosomal protein L38	Ossification	0.00	0.00	391.99	0.00	38.51	1.24 × 10^−4^	1.24 × 10^−4^
6422	SFRP1	Secreted frizzled related protein 1	Ossification; Regulation of ossification	0.00	0.00	14.30	0.00	33.74	2.38 × 10^−4^	4.62 × 10^−2^
10736	SIX2	SIX homeobox 2	Regulation of ossification	0.00	0.00	31.25	0.00	34.86	6.60 × 10^−4^	4.22 × 10^−2^
4086	SMAD1	SMAD family member 1	Ossification; Regulation of ossification	0.00	0.00	45.60	0.00	35.41	2.71 × 10^−3^	2.38 × 10^−4^
6615	SNAI1	Snail family transcriptional repressor 1	Ossification	0.00	0.00	40.55	0.00	35.24	1.24 × 10^−4^	1.24 × 10^−4^
6662	SOX9	SRY-box 9	Ossification; Regulation of ossification	0.00	0.00	16.34	0.00	33.93	1.24 × 10^−4^	1.24 × 10^−4^
169200	TMEM64	Transmembrane protein 64	Ossification; Regulation of ossification	0.00	0.00	13.21	0.00	33.62	1.24 × 10^−4^	3.06 × 10^−3^
10766	TOB2	Transducer of ERBB2, 2	Regulation of ossification	0.00	19.14	27.96	34.16	34.70	1.24 × 10^−4^	1.24 × 10^−4^

3D-PLA: poly-(lactide); CM: conditioned medium; FDR: false discovery rate; GO: gene ontology; hGMSCs: human gingival mesenchymal stem cells.

**Table 2:** Summary of in vivo findings of the different experimental groups.

Experimental Groups	Experimental Procedures	Results
3D-PLA (*N* = 4)	Rats subjected to scraping of the cortical calvaria bone tissue and implant of 3D-PLA	Evaluation 6 weeks after surgery evidenced that ECM not fully covered the interstitial area of the biomaterial porous and empty areas were present inside the biomaterial structure.
3D-PLA + hGMSCs (*N* = 4)	Rats subjected to scraping of the cortical calvaria bone tissue and implant of 3D-PLA enriched with hGMSCs	Evaluation 6 weeks after surgery showed more ECM deposition inside the biomaterial scaffold. At high magnification ECM, without signs of mineralization, is present on the filaments side and at the interface of the native bone and scaffold.
3D-PLA + CM (*N* = 4)	Rats subjected to scraping of the cortical calvaria bone tissue and implant of 3D-PLA enriched with CM	Evaluation 6 weeks after surgery showed a strong ECM deposition inside the biomaterial structure and on the native bone tissue. New blood vessel structure are visible in the ECM and on the interface of scaffold and host tissue, to indicate the beginning of the regeneration process.
3D-PLA + CM + hGMSCs (*N* = 4)	Rats subjected to scraping of the cortical calvaria bone tissue and implant of 3D-PLA enriched with CM and hGMSCs	Evaluation 6 weeks after surgery showed less blood vessels.

PLA: Poly-(lactide); CM: conditioned medium; hGMSCs: human gingival mesenchymal stem cells; ECM: extracellular matrix.

## Abbreviations

PLA	Poly-(lactide)
MSCs	Mesenchymal stem cells
GMSCs	Gingival mesenchymal stem cells
CM	Conditioned medium
SEM	Scanning electron microscopy
hGMSCs	Human gingival mesenchymal stem cells
NGS	Next generation sequencing
GO	Gene ontology
ECM	Extracellular matrix
3D	Three-dimensional
SHED	Stem cells from human exfoliated deciduous teeth
RIPA	Radioimmunoprecipitation assay
ARS	Alizarin Red S
UCSC	University of California, Santa Cruz
FPKM	Fragments per kilobase of exon per million fragments mapped

## References

[1] Florencio-Silva R., Sasso G.R.D.S., Sasso-Cerri E., Simoes M.J., Cerri P.S. (2015). Biology of bone tissue: Structure, function, and factors that influence bone cells. BioMed Res. Int..

[2] Wu T., Yu S., Chen D., Wang Y. (2017). Bionic design, materials and performance of bone tissue scaffolds. Materials.

[3] Roseti L., Parisi V., Petretta M., Cavallo C., Desando G., Bartolotti I., Grigolo B. (2017). Scaffolds for bone tissue engineering: State of the art and new perspectives. Mater. Sci. Eng. C Mater. Biol. Appl..

[4] Tyler B., Gullotti D., Mangraviti A., Utsuki T., Brem H. (2016). Polylactic acid (pla) controlled delivery carriers for biomedical applications. Adv. Drug Deliv. Rev..

[5] Farah S., Anderson D.G., Langer R. (2016). Physical and mechanical properties of pla, and their functions in widespread applications—A comprehensive review. Adv. Drug Deliv. Rev..

[6] Holmes B., Zhu W., Li J., Lee J.D., Zhang L.G. (2015). Development of novel three-dimensional printed scaffolds for osteochondral regeneration. Tissue Eng. Part A.

[7] Gregor A., Filova E., Novak M., Kronek J., Chlup H., Buzgo M., Blahnova V., Lukasova V., Bartos M., Necas A. (2017). Designing of pla scaffolds for bone tissue replacement fabricated by ordinary commercial 3d printer. J. Biol. Eng..

[8] Mushahary D., Spittler A., Kasper C., Weber V., Charwat V. (2017). Isolation, cultivation, and characterization of human mesenchymal stem cells. Cytometry A.

[9] Dominici M., Le Blanc K., Mueller I., Slaper-Cortenbach I., Marini F., Krause D., Deans R., Keating A., Prockop D., Horwitz E. (2006). Minimal criteria for defining multipotent mesenchymal stromal cells. The international society for cellular therapy position statement. Cytotherapy.

[10] Liu J., Yu F., Sun Y., Jiang B., Zhang W., Yang J., Xu G.-T., Liang A., Liu S. (2015). Concise reviews: Characteristics and potential applications of human dental tissue-derived mesenchymal stem cells. Stem Cells.

[11] Fawzy El-Sayed K.M., Dorfer C.E. (2016). Gingival mesenchymal stem/progenitor cells: A unique tissue engineering gem. Stem Cells Int..

[12] Asutay F., Acar H.A., Yolcu U., Kirtay M., Alan H. (2015). Dental stem cell sources and their potentials for bone tissue engineering. J. Istanb. Univ. Fac. Dent..

[13] Wang F., Yu M., Yan X., Wen Y., Zeng Q., Yue W., Yang P., Pei X. (2011). Gingiva-derived mesenchymal stem cell-mediated therapeutic approach for bone tissue regeneration. Stem Cells Dev..

[14] Xu Q.C., Wang Z.G., Ji Q.X., Yu X.B., Xu X.Y., Yuan C.Q., Deng J., Yang P.S. (2014). Systemically transplanted human gingiva-derived mesenchymal stem cells contributing to bone tissue regeneration. Int. J. Clin. Exp. Pathol..

[15] Tomar G.B., Srivastava R.K., Gupta N., Barhanpurkar A.P., Pote S.T., Jhaveri H.M., Mishra G.C., Wani M.R. (2010). Human gingiva-derived mesenchymal stem cells are superior to bone marrow-derived mesenchymal stem cells for cell therapy in regenerative medicine. Biochem. Biophys. Res. Commun..

[16] Tomasello L., Mauceri R., Coppola A., Pitrone M., Pizzo G., Campisi G., Pizzolanti G., Giordano C. (2017). Mesenchymal stem cells derived from inflamed dental pulpal and gingival tissue: A potential application for bone formation. Stem Cell Res. Ther..

[17] Vizoso F.J., Eiro N., Cid S., Schneider J., Perez-Fernandez R. (2017). Mesenchymal stem cell secretome: Toward cell-free therapeutic strategies in regenerative medicine. Int. J. Mol. Sci..

[18] Pawitan J.A. (2014). Prospect of stem cell conditioned medium in regenerative medicine. BioMed Res. Int..

[19] Osugi M., Katagiri W., Yoshimi R., Inukai T., Hibi H., Ueda M. (2012). Conditioned media from mesenchymal stem cells enhanced bone regeneration in rat calvarial bone defects. Tissue Eng. Part A.

[20] Inukai T., Katagiri W., Yoshimi R., Osugi M., Kawai T., Hibi H., Ueda M. (2013). Novel application of stem cell-derived factors for periodontal regeneration. Biochem. Biophys. Res. Commun..

[21] Katagiri W., Osugi M., Kinoshita K., Hibi H. (2015). Conditioned medium from mesenchymal stem cells enhances early bone regeneration after maxillary sinus floor elevation in rabbits. Implant Dent..

[22] Katagiri W., Watanabe J., Toyama N., Osugi M., Sakaguchi K., Hibi H. (2017). Clinical study of bone regeneration by conditioned medium from mesenchymal stem cells after maxillary sinus floor elevation. Implant Dent..

[23] Linero I., Chaparro O. (2014). Paracrine effect of mesenchymal stem cells derived from human adipose tissue in bone regeneration. PLoS ONE.

[24] Zhang B., Zhang P.B., Wang Z.L., Lyu Z.W., Wu H. (2017). Tissue-engineered composite scaffold of poly(lactide-co-glycolide) and hydroxyapatite nanoparticles seeded with autologous mesenchymal stem cells for bone regeneration. J. Zhejiang Univ. Sci. B.

[25] Guvendiren M., Molde J., Soares R.M., Kohn J. (2016). Designing biomaterials for 3d printing. ACS Biomater. Sci. Eng..

[26] Liu A., Xue G.H., Sun M., Shao H.F., Ma C.Y., Gao Q., Gou Z.R., Yan S.G., Liu Y.M., He Y. (2016). 3D printing surgical implants at the clinic: A experimental study on anterior cruciate ligament reconstruction. Sci. Rep..

[27] Gremare A., Guduric V., Bareille R., Heroguez V., Latour S., L’Heureux N., Fricain J.C., Catros S., Le Nihouannen D. (2017). Characterization of printed pla scaffolds for bone tissue engineering. J. Biomed. Mater. Res. Part A.

[28] Tran C., Damaser M.S. (2015). Stem cells as drug delivery methods: Application of stem cell secretome for regeneration. Adv. Drug Deliv. Rev..

[29] Noiseux N., Gnecchi M., Lopez-Ilasaca M., Zhang L., Solomon S.D., Deb A., Dzau V.J., Pratt R.E. (2006). Mesenchymal stem cells overexpressing akt dramatically repair infarcted myocardium and improve cardiac function despite infrequent cellular fusion or differentiation. Mol. Ther. J. Am. Soc. Gene Ther..

[30] Libro R., Diomede F., Scionti D., Piattelli A., Grassi G., Pollastro F., Bramanti P., Mazzon E., Trubiani O. (2016). Cannabidiol modulates the expression of Alzheimer’s disease-related genes in mesenchymal stem cells. Int. J. Mol. Sci..

[31] Wada N., Maeda H., Hasegawa D., Gronthos S., Bartold P.M., Menicanin D., Fujii S., Yoshida S., Tomokiyo A., Monnouchi S. (2014). Semaphorin 3a induces mesenchymal-stem-like properties in human periodontal ligament cells. Stem Cells Dev..

[32] Wang X., Li G., Liu Y., Yu W., Sun Q. (2017). Biocompatibility of biological material polylactic acid with stem cells from human exfoliated deciduous teeth. Biomed. Rep..

[33] Timashev P., Kuznetsova D., Koroleva A., Prodanets N., Deiwick A., Piskun Y., Bardakova K., Dzhoyashvili N., Kostjuk S., Zagaynova E. (2016). Novel biodegradable star-shaped polylactide scaffolds for bone regeneration fabricated by two-photon polymerization. Nanomedicine.

[34] Rajan T.S., Giacoppo S., Diomede F., Ballerini P., Paolantonio M., Marchisio M., Piattelli A., Bramanti P., Mazzon E., Trubiani O. (2016). The secretome of periodontal ligament stem cells from ms patients protects against eae. Sci. Rep..

[35] Ekwueme E.C., Shah J.V., Mohiuddin M., Ghebes C.A., Crispim J.F., Saris D.B.F., Fernandes H.A.M., Freeman J.W. (2016). Cross-talk between human tenocytes and bone marrow stromal cells potentiates extracellular matrix remodeling in vitro. J. Cell. Biochem..

[36] Ando Y., Matsubara K., Ishikawa J., Fujio M., Shohara R., Hibi H., Ueda M., Yamamoto A. (2014). Stem cell-conditioned medium accelerates distraction osteogenesis through multiple regenerative mechanisms. Bone.

[37] Collett G.D.M., Canfield A.E. (2005). Angiogenesis and pericytes in the initiation of ectopic calcification. Circ. Res..

[38] Diomede F., Rajan T.S., Gatta V., D’Aurora M., Merciaro I., Marchisio M., Muttini A., Caputi S., Bramanti P., Mazzon E. (2017). Stemness maintenance properties in human oral stem cells after long-term passage. Stem Cells Int..

[39] Rajan T.S., Scionti D., Diomede F., Grassi G., Pollastro F., Piattelli A., Cocco L., Bramanti P., Mazzon E., Trubiani O. (2017). Gingival stromal cells as an in vitro model: Cannabidiol modulates genes linked with amyotrophic lateral sclerosis. J. Cell. Biochem..

[40] Libro R., Scionti D., Diomede F., Marchisio M., Grassi G., Pollastro F., Piattelli A., Bramanti P., Mazzon E., Trubiani O. (2016). Cannabidiol modulates the immunophenotype and inhibits the activation of the inflammasome in human gingival mesenchymal stem cells. Front. Phys..

[41] Ballerini P., Diomede F., Petragnani N., Cicchitti S., Merciaro I., Cavalcanti M., Trubiani O. (2017). Conditioned medium from relapsing-remitting multiple sclerosis patients reduces the expression and release of inflammatory cytokines induced by lps-gingivalis in thp-1 and mo3.13 cell lines. Cytokine.

[42] Gugliandolo A., Diomede F., Cardelli P., Bramanti A., Scionti D., Bramanti P., Trubiani O., Mazzon E. (2017). Transcriptomic analysis of gingival mesenchymal stem cells cultured on 3D bioprinted scaffold: A promising strategy for neuroregeneration. J. Biomed. Mater. Res. Part A.

[43] Diomede F., Caputi S., Merciaro I., Frisone S., D’Arcangelo C., Piattelli A., Trubiani O. (2014). Pro-inflammatory cytokine release and cell growth inhibition in primary human oral cells after exposure to endodontic sealer. Int. Endod. J..

[44] Rajan T.S., Giacoppo S., Trubiani O., Diomede F., Piattelli A., Bramanti P., Mazzon E. (2016). Conditioned medium of periodontal ligament mesenchymal stem cells exert anti-inflammatory effects in lipopolysaccharide-activated mouse motoneurons. Exp. Cell Res..

[45] Diomede F., Zini N., Gatta V., Fulle S., Merciaro I., D’Aurora M., La Rovere R.M., Traini T., Pizzicannella J., Ballerini P. (2016). Human periodontal ligament stem cells cultured onto cortico-cancellous scaffold drive bone regenerative process. Eur. Cells Mater..

